# Endogenous Antioxidant Cocktail Loaded Hydrogel for Topical Wound Healing of Burns

**DOI:** 10.3390/pharmaceutics13010008

**Published:** 2020-12-22

**Authors:** José L. Soriano, Ana C. Calpena, María J. Rodríguez-Lagunas, Òscar Domènech, Nuria Bozal-de Febrer, María L. Garduño-Ramírez, Beatriz Clares

**Affiliations:** 1 Department of Pharmacy and Pharmaceutical Technology, Faculty of Pharmacy, University of Granada, 18071 Granada, Spain; jlsoriano@correo.ugr.es; 2 Department of Pharmacy and Pharmaceutical Technology and Physical Chemistry, Faculty of Pharmacy and Food Sciences, University of Barcelona, 08028 Barcelona, Spain; odomenech@ub.edu; 3 Nanoscience & Nanotechnology Institute (IN2UB), University of Barcelona, 08028 Barcelona, Spain; 4 Department of Biochemistry and Physiology, Faculty of Pharmacy and Food Sciences, University of Barcelona, 08028 Barcelona, Spain; mjrodriguez@ub.edu; 5 Nutrition and Food Safety Research Institute (INSA-UB), 08921 Santa Coloma de Gramenet, Spain; 6 Department of Biology, Healthcare and the Environment, Faculty of Pharmacy and Food Sciences, University of Barcelona, 27-31 Joan XXIII Ave., 08028 Barcelona, Spain; nuriabozaldefebrer@ub.edu; 7 Centro de Investigaciones Químicas, Universidad Autónoma del Estado de Morelos, Av. Universidad No. 1001, Col Chamilpa, 62209 Cuernavaca, Mexico; lgarduno@ciq.uaem.mx; 8 Biosanitary Institute of Granada (ibs.GRANADA), 18012 Granada, Spain

**Keywords:** vitamins, melatonin, antioxidant, skin, healing, ATF microscopy

## Abstract

The main goal of this work is the study of the skin wound healing efficacy of an antioxidant cocktail consisting of vitamins A, D, E and the endogenous pineal hormone melatonin (MLT), with all of these loaded into a thermosensitive hydrogel delivery system. The resulting formulation was characterized by scanning electron microscopy. The antioxidant efficacy and microbiological activity against Gram positive and Gram negative strains were also assayed. The skin healing efficacy was tested using an in vivo model which included histological evaluation. Furthermore, atomic force microscopy was employed to evaluate the wound healing efficacy of rat skin burns through the determination of its elasticity at the nanoscale using force spectroscopy analysis. The resulting hydrogel exhibited sol state at low temperature and turned into a gel at 30 ± 0.2 °C. The hydrogel containing the antioxidant cocktail showed higher scavenging activity than the hydrogel containing vitamins or MLT, separately. The formulation showed optimal antimicrobial activity. It was comparable to a commercial reference. It was also evidenced that the hydrogel containing the antioxidant cocktail exhibited the strongest healing process in the skin burns of rats, similar to the assayed commercial reference containing silver sulfadiazine. Histological studies confirmed the observed results. Finally, atomic force microscopy demonstrated a similar distribution of Young’s modulus values between burned skin treated with the commercial reference and burned skin treated with hydrogel containing the antioxidant cocktail, and all these with healthy skin. The use of an antioxidant cocktail of vitamins and MLT might be a promising treatment for skin wounds for future clinical studies.

## 1. Introduction

The skin is a microbiological, chemical, immunological and physical barrier that confers protection to the organism against external harms [1]. This barrier can be disrupted, allowing external pathogens to enter the body, which causes inflammation and infection. Among skin wounds, burns are the most frequent skin injuries. This kind of injury can be caused by thermal, electrical, chemical, or electromagnetic energy. The severity of the burn correlates with the layers of the epidermis, dermis and hypodermis affected, increasing the morbidity and mortality when the surface area of the burn increases [2]. The induced damage is accompanied by the activation of inflammatory and coagulation processes, as well as an excess of cytotoxic reactive oxygen and nitrogen species (ROS, RNS), involving secondary tissue damage [3]. In this regard, the development of effective antioxidant tools for cutaneous wound healing would help to reduce tissue damage and wound infection, as well as other associated complications [4]. Recently, this antioxidant strategy has been also investigated [5,6]. Classical treatments of topical wound burns include the gold standard silver sulfadiazine. However, some detrimental effects such as cytotoxic activity and wound healing delay have been described [7]. Thus, the research of new approaches for controlling infection and avoiding undesired cytotoxic effects, at the same time, represents a challenge of paramount importance nowadays. Among antioxidant agents for wound healing, several types of vitamins have been proposed, such as vitamins E (alpha tocopherol) and D. Alpha tocopherol prevents the oxidation damage of collagen and glycosaminoglycans during wound healing, being able to accelerate the wound closure and ameliorate burn infection [8,9]. Regarding vitamin D, this vitamin has been reported to modulate immune responses [10], and it has also been proved to present a beneficial effect in sun burns [11]. Moreover, vitamin D triggers the formation of antimicrobial peptides that may contribute to the wound healing process [12]. Vitamin A, despite not being a common antioxidant, is able to reduce oxidative stress [13] and even prevent the cancer risks of oxidative damage [14]. Recent studies have shown that vitamin A favors superoxide dismutase and glutathione transferase activities [15]. Furthermore, it has also been associated with participation in wound healing, specifically in macrophage-mediated inflammatory processes as well as angiogenesis [16]. Taking all these vitamins together, we have previously developed a thermosensitive hydrogel loaded with vitamin A, D and E for the treatment of skin burns [17].

Other antioxidant molecules, such as the endogenous hormone melatonin (MLT; 5-methoxy-N-acetyl-tryptamine), has also been assayed in this field [18]. MLT is secreted by the pineal gland and plays an important role as a potent radical scavenger of reactive oxygen species (ROS) and reactive nitrogen species (RNS). In a previous paper, we reviewed the role of melatonin (MLT) as a powerful antioxidant [19]. The antioxidant effects of MLT occur through direct and indirect mechanisms. On one hand, MLT scavenges free radicals in all compartments of the body due to its amphiphilia and its distribution capacity [20]. MLT has been shown to detoxify up to 10 radicals [21]. On the other hand, MLT increases the activity of antioxidant enzymes. This causes an increase in the endogenous antioxidant defense capacity and induces the upregulation of gene expression, increasing the first line of defense against oxidative damage to cells [22]. Due to these properties, it has been proposed as a potential treatment for wound healing [23,24], being more effective than vitamins C and E [25,26]. In addition to the anti-inflammatory effect of MLT, it has some advantages with respect to other antioxidant molecules, such as its endogenous nature and its facility to enter into subcellular compartments, among others [19,27]. Among the beneficial effects of MLT, in vitro and in vivo antimicrobial properties have been also reported [28,29]. In this sense, MLT has shown activity against fungi such as *Sacchoramyces cerevisae* or *Candida albicans* [30], some viruses [31] and both Gram positive and Gram negative bacteria. Some authors have concluded that its antimicrobial activity could be due to the reduction in intracellular substrates, which leads to a prolongation of the lag phase of bacterial growth [32].

Thus, the possibility of using vitamins and MLT together might be a suitable combination for treating skin burns and improving the proliferation and differentiation of skin cells. For this task, a drug-delivery system for skin wound healing able to absorb wound exudates and provide a moist environment with intrinsic antimicrobial properties should be desirable. Different drug-delivery systems for loading these antioxidant drugs have been developed so far for treating wounds, such as microspheres [33], nanoparticles [34], nanospheres [35], etc. It is also remarkable to take into consideration the biological properties of biomaterials forming the drug carrier. In previous studies, our group has demonstrated the potential of a hydrogel loading antioxidant actives in wound healing, consisting of poloxamer 407 (PLX), chitosan (CS) and hyaluronic acid (HA) [17,36]. Hydrogel might absorb exudates and provide a moist environment, preventing wound dehydration [37]. In this context, in situ gel-forming hydrogel is even more appropriate due to its ability to fill the wound area as sol state just after topical administration and adhere when converted into gel [38].

As a next step, it is our intention to evaluate the effect of antioxidant actives, vitamins and MLT together, as well as the use of techniques that help to record the healing action of these antioxidant drugs on the skin.

Atomic Force Microscopy (AFM) has emerged as a useful technique not only to visualize samples at a nanometric level, but also as a powerful technique to quantify functional and structural characteristics from the nanoscale to the microscale, from single molecules to whole cells and tissues. AFM has been widely used to investigate individual cells [39] or the extracellular matrix (ECM) of soft tissues [40,41]. In recent years, the investigation of the whole tissue [42,43] is an emerging field to understand, in a bottom-up way, the classical biological experiments on tissues.

In the present work, we proposed a strategy for the wound healing of burns, which combined antioxidant vitamins A, E, D and the endogenous hormone MLT. The antioxidant cocktail was loaded in a PLX/CS/HA hydrogel. For this task, the wound healing efficacy was assessed in vivo and histologically. Furthermore, we evaluated the elastic modulus, Young’s modulus, by means of force spectroscopy AFM (FS-AFM) of burned rat skin samples after different treatments to investigate the therapeutic goodness on a cutaneous wound model. In order to support the previous tests, the antibacterial and antioxidant activity was also determined.

## 2. Materials and Methods

### 2.1. Materials and Animals

MLT and sodium hyaluronate (1.46 MDa) were purchased from Acofar (Madrid, Spain). Retinol palmitate (vitamin A) 200,000 IU/mL, cholecalciferol (vitamin D3) 100,000 IU/g, and D,L alpha tocopherol acetate (vitamin E) were supplied by Fagron Iberica (Barcelona, Spain). PLX was obtained from BASF (Barcelona, Spain). Sigma-Aldrich (Madrid, Spain) supplied 75–85% deacetylated CS (190–310 KDa).

The commercial topical cream silvederma^®^ containing silver sulfadiazine 10 mg/g (Aldo-Union Co., Barcelona, Spain) expiry date 01/2021, batch 0012M003, was purchased from a local pharmacy. Double distilled water was obtained from a device Milli-Q^®^ Gradient A10 (Millipore Iberica, Madrid, Spain). All other materials used in experimental sections were of analytical grade and supplied by Sigma-Aldrich (Madrid, Spain) unless otherwise specified.

Wistar rats, 3–5 months old and weighing 300–500 g, were purchased from the Laboratory Animal Center of Barcelona University. The animals were maintained under standard conditions with free access to food and water in the laboratory animal facilities for one week before the beginning of studies. All rats were treated humanely and under veterinary supervision throughout the experimental period.

### 2.2. Preparation and Characterization of Hydrogel

The PLX/CS/HA based hydrogel was prepared via wet conventional synthesis by direct dispersion in water. Briefly, 0.2% HA solution (*w*/*v*) was prepared by adding the required amount of HA to double distilled water and then stirred for 1 h, subsequently the solution was filtered. Then, a 0.5 % (*w*/*v*) CS/acetic acid solution was obtained by dispersing the correct amount of CS in 0.5% acetic acid. Equally, 1.8% PLX (*w*/*v*) was dissolved in double distilled water with continuous stirring. The inclusion of actives was carried out as follows.

A pre-selected amount of vitamin D was poured into the required volumes of vitamins A and E and dispersed. This mixture, together with the amount of MLT required to reach 1% (*w*/*v*), was added to the previous PLX solution until solubilization at 4 °C. Afterwards, this solution containing actives and the CS solution were added to the HA solution at 4 °C and stirred continuously for 24 h. Finally, the required amount of PLX to reach 18% *(w*/*v*) was added, and this was left to stand for 24 h. The final composition of the formulation labeled as PLX/CS/HA-VM was: PLX 18% (*w*/*v*), CS 0.5% (*w*/*v*), HA 0.2% (*w*/*v*), vitamin A 6000 IU/g, vitamin D 400 IU/g, vitamin E 2% (*w*/*v*) and MLT 1% (*w*/*v*). Furthermore, PLX/CS/HA hydrogel loading vitamins (PLX/CS/HA-V) or MLT (PLX/CS/HA-M) were also prepared for antioxidant, histological and AFM studies comparisons.

The sol/gel transition temperature and time was assessed in triplicate by the measurement of time and temperature at which a magnetic bar stopped moving owing to gelation. For this task, a 10 mL sample was put into a transparent vial containing the magnetic bar at constant rotation speed of 400 rpm in a magnetic water bath from 4 to 37 ± 0.1 °C.

Scanning electron microscopy (FEI Quanta^®^ FEG 650, Thermo Fisher Scientific-FEI, Hillsboro, CA, USA) was utilized to investigate the morphology of PLX/CS/HA-VM. Samples were processed using the critical point drying technique. PLX/CS/HA-VM was sputtered with carbon before observation.

The pH value of hydrogel was recorded using a digital pH meter GLP 22 (Crison Instruments, Alella, Spain) at room temperature. The measurements were conducted by direct immersion of the device electrode in the sample. Obtained data are expressed as the mean ± SD of three replicates.

### 2.3. Antioxidant Efficiency

The radical scavenging activity of blank PLX/CS/HA, PLX/CS/HA-V, PLC/CS/HA-M and PLX/CS/HA-VM was tested by measuring their capacity to scavenge the stable 1, 1-diphenyl-2-picrylhydrazyl (DPPH, Sigma-Aldrich Chemie, Steinheim, Germany). For this task, each hydrogel was prepared by taking 500 µL of the formulation and 1500 µL of the DPPH ethanolic solution. The reaction mixtures were shaken vigorously and then kept at 30 °C. Their absorbances were recorded in triplicate spectrophotometrically with the method of a slightly modified test of Brand-Williams [44] at 515 nm on a Spectronic Genesys 8 UV/Vis spectrophotometer (Fisher Scientific, Rochester, NJ, USA).

The percentage of radical scavenging activity (RSA%) was calculated according to the following equation:

(1) $$\mathrm{RSA}\%=\frac{A{\;}_{0}-A{\;}_{s}}{A{\;}_{0}}\times 100$$

where A_0_ is the absorbance of the control and A_s_ is the absorbance of the samples at 515 nm.

### 2.4. Antimicrobial Activity

The antimicrobial activity test was carried out by the Kirby-Bauer Disk Diffusion Susceptibility Test [45]. Gram negative bacteria such as *Acinetobacter baumanii* ATCC 19606, *A. baumanii* ABAU clinically isolated, *Escherichia coli* ATCC 25922, *Pseudomonas aeruginosa* ATCC 27823 and *P. aeruginosa* PAO-1 clinically isolated, as well as Gram positive bacteria such as *Staphylococcus aureus* ATCC 29213 and *Staphylococcus aureus* MARSA ATCC 43300) and fungi Candida albicans ATCC10231 were assayed. MacFarland standard suspensions (1.5 × 108 colony-forming units/mL) were used for each strain and then added on Mueller-Hinton agar plates. Then, sterile filter-paper discs (6 mm diameter) were placed on the surface and 25 μL of a sample was placed onto the discs. The agar plates were incubated at 37 °C for 48 h.

### 2.5. In Vivo Wound Healing Study

This study was previously approved by the animal research ethical committee 387/18 of the University of Barcelona according to Spanish law (Royal Decree 53/1 February 2013) based on the European directive 2010/63/UE.

The animals were anesthetized by isoflurane 0.5% at 5 L/min administration followed by an intradermal injection of Buprex^®^ 0.05 mg/kg analgesic. The animals were supervised by a veterinarian throughout the study.

Firstly, the backs of the animals were shaved and cleaned with 70% ethanol. The rats were divided into groups of three individuals (*n* = 3). Skin burns were caused by a 1.0 cm^2^ superficial contact area of cylindrical devices at 100 °C. The treatments were applied once daily for two weeks in the following way: Group I positive control, the animals in this group received no treatment; Group II, the animals were treated with 100 µL of PLX/CS/HA without antioxidant actives; Group III, the animals were treated with 100 µL of PLX/CS/HA-VM; Group IV, the animals were treated with the commercial reference silvederma®. The wound healing process (WH) was recorded by the measurement of the wound size and applying the following equation:

WH(%) = (initial wound size-final wound size)/(initial wound size) × 100(2)

### 2.6. Histological Analysis

After the burns were induced, the study animals were euthanized by cervical dislocation. The skin of the burned areas was excised. The tissues were fixed for 24 h at 25 °C in formaldehyde and then washed with a phosphate buffered solution (PBS). The samples were then dehydrated by immersion in ascending grades of ethanol solutions and cleared with xylene. Once the samples were dehydrated, they were embedded in paraffin. To proceed with visualization, the samples were cut into 5 µm sections, stained with hematoxylin/eosin and mounted in a light microscope Olympus BX41 equipped with an Olympus XC50 camera (Olympus Co., Tokyo, Japan). A neutrophil quantification was carried out, which involved dividing samples into three parts and counting in three random foci of the dermis.

### 2.7. AFM Force Spectroscopy Experiments

For the AFM experiments, each skin sample was defrosted at room temperature and cut by dermatome (Model GA 630, Aesculap, Tuttlingen, Germany) into 400 μm thick pieces. After that, the 0.5 × 0.5 cm^2^ pieces of rat skin were immediately glued onto a steel disc and rinsed gently with buffer and with deionized water to eliminate any surface contaminant. Finally, the sample surface was dried under a nitrogen stream. Each sample was mounted directly onto the AFM scanner located in a chamber with a controlled temperature and humidity to prevent water loss from the skin at 24 °C and 70%, respectively.

The AFM device consisted of an AFM Multimode IV controlled by Nanoscope V electronics (Bruker AXS Corporation; Santa Barbara, CA) equipped with a 15 µm piezoelectric scanner. Silicon AFM tips with a nominal spring constant of 42 nN nm^−1^ were used. The samples were studied in contact mode and in air, with a scan rate of 1.5 Hz and a scan angle of 0°.

For AFM Force Spectroscopy measurements, the spring constant of each cantilever used was determined using the thermal noise method. Hundreds of forces versus tip–sample distance curves were acquired in at least 10 different spots of each sample. The tip–sample approaching velocity was set for all force curves at 1000 nm s^−1^. Applied forces F are given by F = kc × Δ, where kc is the spring constant of the cantilever and Δ stands for the cantilever deflection. The surface deformation is given as penetration (δ), evaluated as δ = z − Δ, where z represents the piezo-scanner displacement.

On the other hand, the rat skin elastic modulus (Young’s modulus) was calculated using the Hertz model by converting force versus tip–sample distance curves into force versus indentation curves. The Hertz model is a good approximation to our system as it assumes indentation is negligible in comparison to the sample thickness, so that the substrate does not influence the calculations. Thus, experimental force versus δ data were adjusted to Equation (3) to obtain the Young′s modulus [46].

F = E/(1 − υ2)× tanα/√2 × δ2(3)

where E is the Young’s modulus, α is the half angle of the AFM tip and υ is the so-called Poisson’s ratio. In our experiments, α was 22.5° and υ was assumed to be 0.4 for a slightly compressive surface such as skin.

### 2.8. Statistical Analysis

The results were analyzed with one-way analysis of variance (ANOVA) to evaluate differences among mean values. Prism^®^ software, v. 3.0 (GraphPad Software, Inc., San Diego, CA, USA) was used. A *p*-value < 0.05 was considered statistically significant.

## 3. Results

### 3.1. Characterization

**Figure 1:**
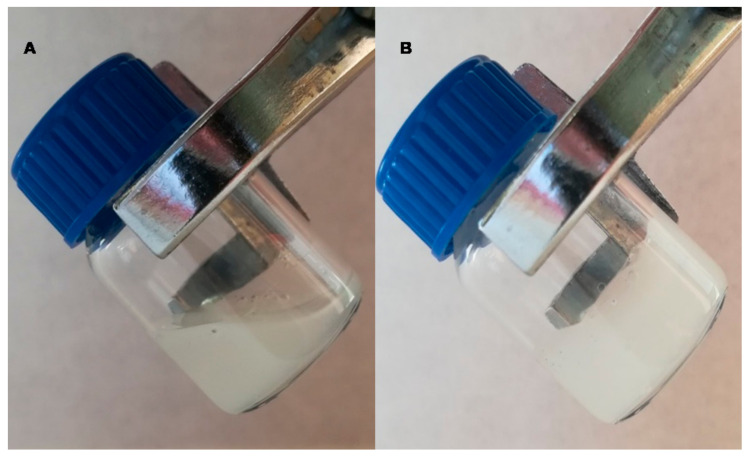
(**A**) PLX/CS/HA-VM at 18 °C; (**B**) PLX/CS/HA-VM at 32 °C.

The developed hydrogel showed a whitish hue and a homogeneous and fluid appearance, with a temperature-dependent sol-gel transition at 30 ± 0.2 °C after 1.7 ± 0.1 min. The hydrogel was a free-flowing sol at low temperature (measurements started from 18 °C) and turned into a non-flowing gel above 30 °C (Figure 1). The obtained pH of PLX/CS/HA-VM was 5.0 ± 0.1.

**Figure 2:**
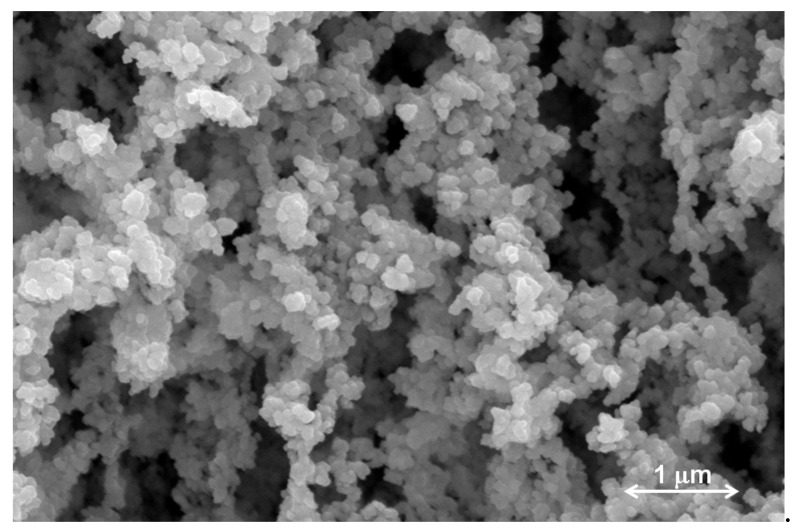
Scanning Electron Microscopy image obtained from PLX/CS/HA-VM, 30,000× magnification.

The internal morphology of PLX/CS/HA-VM was observed by SEM. Figure 2 shows the internal porous three-dimension structure with approximately spherical and homogeneous micellar size.

### 3.2. Antioxidant Activity

**Figure 3:**
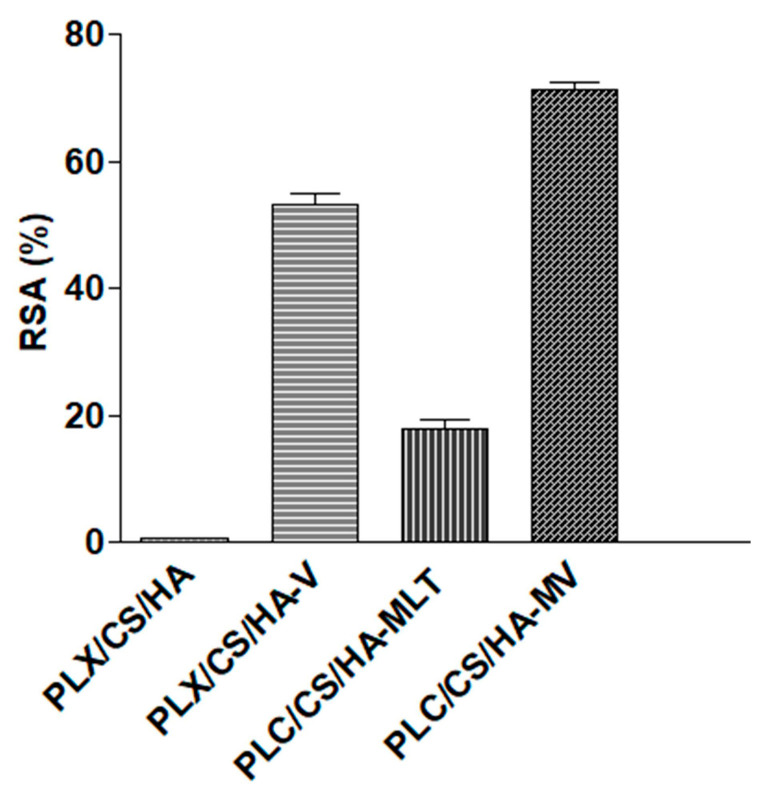
Antioxidant activity of hydrogels.

Figure 3 depicts the antioxidant efficacy results of the hydrogel loading vitamins, MLT and the antioxidant cocktail (vitamins A, D, E and MLT). As expected, blank hydrogel PLX/CS/HA showed no radical scavenging activity. Regarding MLT and vitamins, statistically significant differences were observed between both formulations, with RSA values ⁓18% and ⁓52%, respectively. However, the values of RSA for PLX/CS/HA-VM were significantly higher, in the vicinity of 70%.

### 3.3. Microbiological Studies

Table 1 depicts the microbiological results of PLX/CS/HA-VM and the commercial reference against Gram positive, Gram negative and fungi strains. It is clear that PLX/CS/HA and PLX/CS/HA-VM produced significant inhibition of bacterial growth; they were roughly similar to the commercial reference, except in the case of *E. coli* ATCC 25922, for which both the blank hydrogel and the loaded hydrogel did not show antimicrobial action. Similar results were obtained in the case of growth inhibition assay.

### 3.4. Wound Healing Effect on Rat Skin

**Figure 4:**
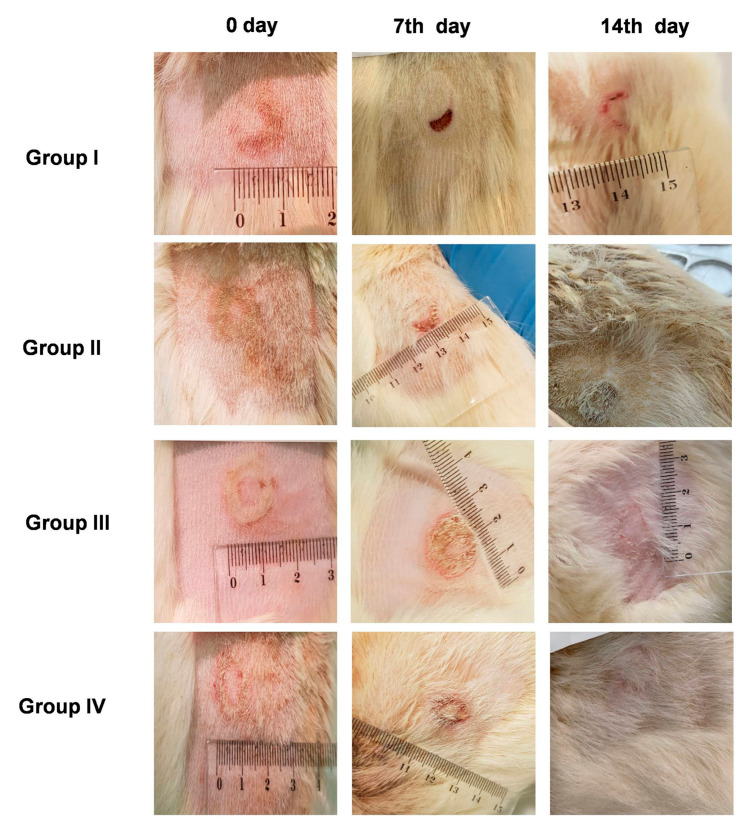
Wound healing evolution for 14 days in animals with no treatment (Group I), treated with PLX/CS/HA (Group II), treated with PLX/CS/HA-VM (Group III), and animals treated with the commercial reference silvederma^®^ (Group IV).

The wound healing efficacy induced by PLX/CS/HA-VM on rat skin burns is shown in Figure 4. Wounds treated with PLX/CS/HA-VM exhibited similar healing progress to those treated with the commercial reference silvederma^®^. After 14 days, a significant acceleration in wound healing was observed as compared with animals treated with the unloaded PLX/CS/HA hydrogel and the untreated group.

On the other hand, wound closure was analyzed in each group as a percentage of the reduction in the wounded area after 14 days (Equation (2)), obtaining the following rates: 54.23% in the case of the untreated group, 63.52% in the case of animals treated with blank hydrogel, 96.12% for the group treated with PLX/CS/HA-VM, and 98.53% for the group treated with the commercial reference.

### 3.5. Histological Observation

**Figure 5:**
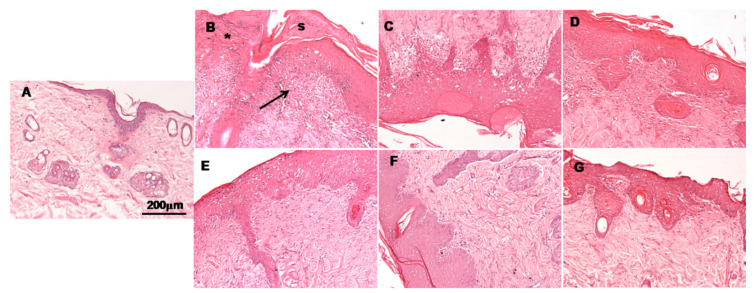
Histology of skin; (**A**) healthy skin; (**B**) burned skin without treatment; (**C**) skin treated with PLX/CS/HA; (**D**) skin treated with PLX/CS/HA-V; (**E**) skin treated with PLX/CS/HA-M; (**F**) skin treated with commercial reference; (**G**) skin treated with PLX/CS/HA-VM. Hematoxylin and eosin stains nuclei blue/black while keratin and cytoplasm are stained red. The asterisk indicates loss of stratum corneum and the arrow indicates infiltration of inflammatory cells, s = scar. Scale bar = 200 μm.

Histological images of burn sites 14 days post-burning are shown in Figure 5. These observations were used to evaluate the healing potential of assayed formulations. In the case of untreated animals, an ulcer and the presence of inflammatory cells covered by a scab could be observed (Figure 5B). The tissue also showed increased epidermal thickness and a loss of epidermal appendices such as hair follicles. A similar pattern was observed in skin samples treated with blank hydrogel (Figure 5B), although the scabs were smaller. In specimens treated with the commercial reference silvederma^®^ (Figure 5F), epidermis growth showing wound contraction could be observed. The groups treated with vitamin loaded hydrogel and MLT loaded hydrogel (Figure 5E,D, respectively) also showed a good recovery, similar to the commercial reference group. However, it seems that in conjunction they could ameliorate the regeneration of the epidermis and dermis, as it can be observed from the histological evaluation of samples treated with PLX/CS/HA-VM, in which both groups of antioxidant actives were included (Figure 5G). In this last case, skin showed less infiltration of inflammatory cells, smaller epidermal thickness and more epidermal appendices when compared to the commercial reference treatment.

### 3.6. Atomic Force Microscopy

**Figure 6:**
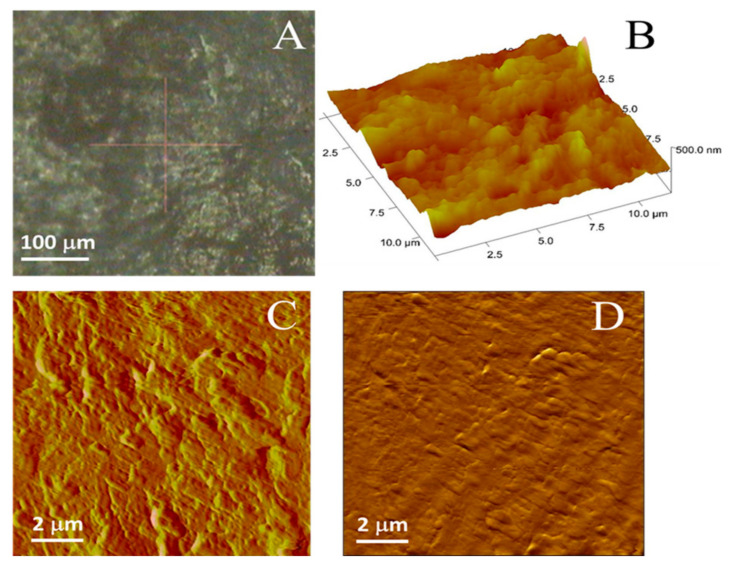
(**A**) Representative images for healthy rat skin as seen in the optical microscope; (**B**) 3D Atomic Force Microscopy (AFM) topographic view; (**C**) AFM deflection error image; (**D**) AFM friction image.

Figure 6 shows representative images of the health of rat skin under study. Figure 6A shows an optical image of clean regions without any detached scales or hair. Brighter zones were preferred over darker ones (intercellular gaps) to avoid any border effect. Figure 6A shows the three-dimensional (3D) topographic view of rat skin surface corresponding to one of the brighter areas displayed in Figure 6A. Some random structures ⁓500 nm wide with mean height values of 50–100 nm can be observed, but no keratin fibrils or organized structures were detected. As the scan size of this image is ⁓15 × 15 µm^2^, the picture is equivalent to one fraction of the surface of a corneocyte. Furthermore, Figure 6A shows the corresponding deflection AFM image derived from Figure 6B. This image highlights the border of the scanned structures and reveals the roughness due to corneocyte disposition typical of healthy skin [47]. Furthermore, the friction AFM image of skin is shown in Figure 6D to assure the cleanliness of the evaluated sample, from which it can be observed that no changes in color are recorded. This is indicative that no lipids or other contaminants are present in the surface of the skin under study.

#### Young’s Modulus from AFM Force Spectroscopy

**Figure 7:**
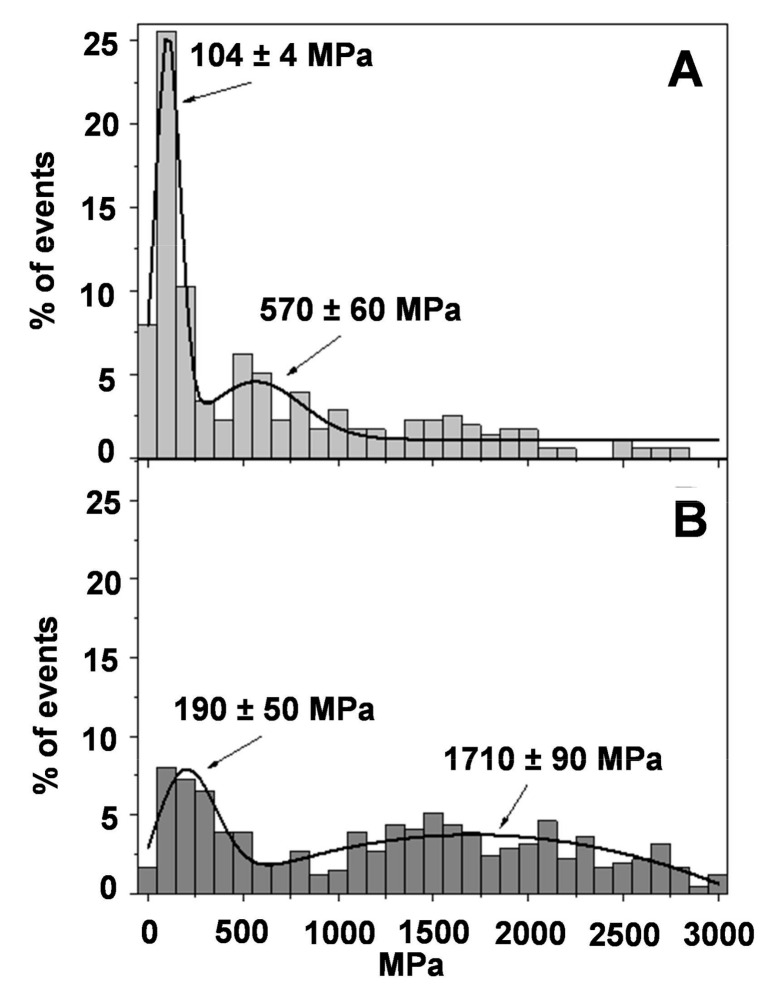
Young’s moduli distribution obtained by means of AFM Force Spectroscopy; (**A**) healthy skin; (**B**) burned skin. Continuous line is the fitting of the experimental data to normal distributions.

Before obtaining force–distance curves to evaluate the Young’s modulus, each sample was visualized to assure a 5 × 5 µm^2^ homogeneous flat area, without contaminants or unattached flakes. Figure 7 shows histograms of the Young’s modulus magnitude for healthy skin (Figure 7A) and burned skin without treatment (Figure 7B). It can clearly be seen that healthy skin presents a maximum (25%) at 104 ± 4 MPa, as expected, indicative of healthy rat skin presenting the highest Young’s modulus value in the range of 0–200 MPa. It is also possible to observe a second peak at 550 MPa, but it is much less intense and diffuse, with frequency values around 5%. These Young’s modulus values are quite high when compared with cells or tissues. In these, Young’s modulus values are around 10–100 kPa [48]. However, the stratum corneum is more rigid than individual cells or tissues, and our values are in agreement with those reported by other similar studies with skin [49].

On the contrary, when the rat skin is damaged (Figure 7B), the maximum observed in the natural skin has almost vanished together with a shift towards higher values (190 MPa). The second peak in Figure 7B is also shifted toward higher Young’s modulus values and spread in a wider range of values. Although it is difficult to attribute the peak, the mathematical fitting of the data to a normal distribution centers the peak at 1700 MPa.

**Figure 8:**
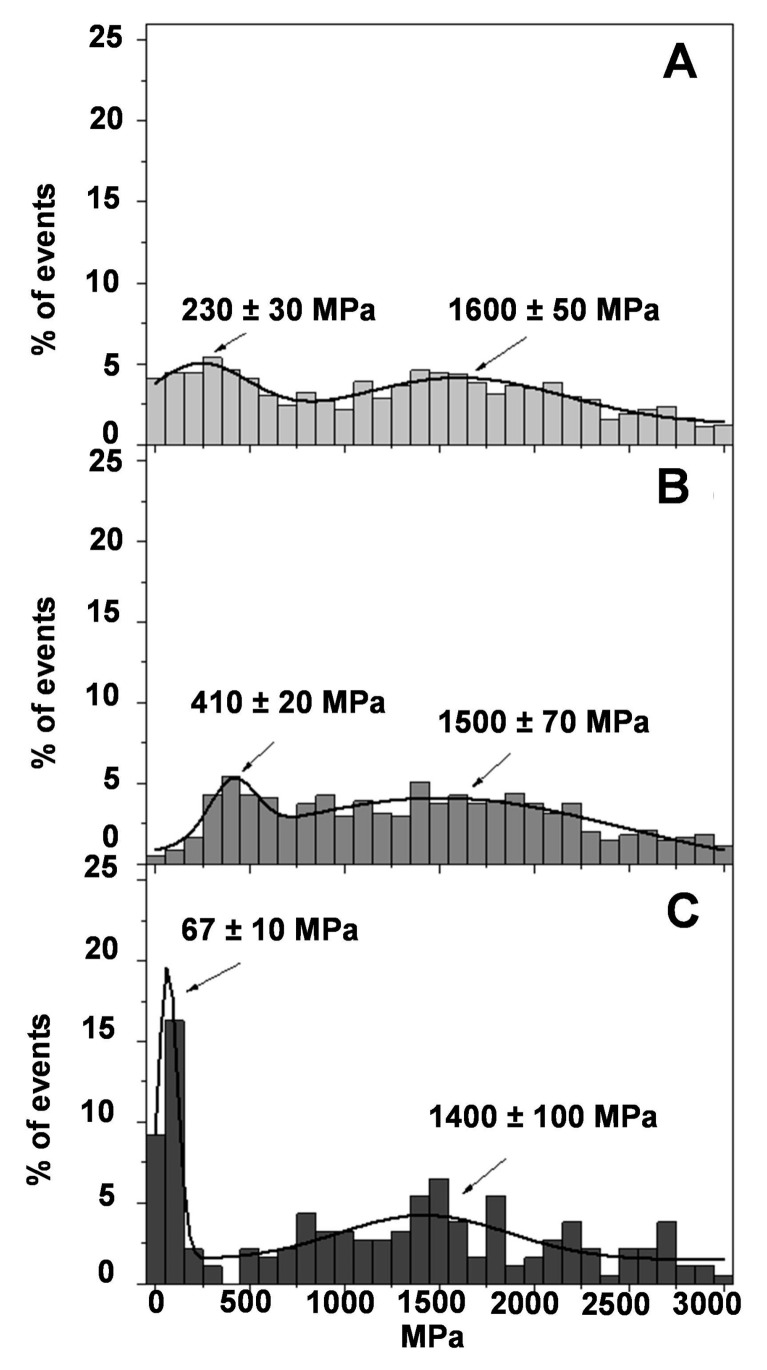
Young’s moduli distribution obtained by means of AFM Force Spectroscopy; (**A**) burned skin treated with PLX/CS/HA; (**B**) burned skin treated with PLX/CS/HA-V; (**C**) burned skin treated with PLX/CS/HA-M. Continuous line is the fitting of the experimental data to normal distributions.

The mechanism in which the stiffness of the damaged rat skin was modified after some treatments was also evaluated. Figure 8 shows histograms of the Young’s modulus for blank hydrogel PLX/CS/HA (Figure 8A), PLX/CS/HA-V (Figure 8B) and PLX/CS/HA-M (Figure 8C). As expected, the treatment with PLX/CS/HA did not significantly modify the Young’s modulus distribution when compared with burned skin (Figure 7B). Surprisingly, the PLX/CS/HA-V, although it has demonstrated some wound healing efficacy in previous studies [17], did not differ significantly from the untreated damaged skin (Figure 7B). Interestingly, the treatment with PLX/CS/HA-M (Figure 8C) partially recovers the main peak found in the normal skin (Figure 7A) but with less frequency, as there are still some regions with high Young’s modulus values.

**Figure 9:**
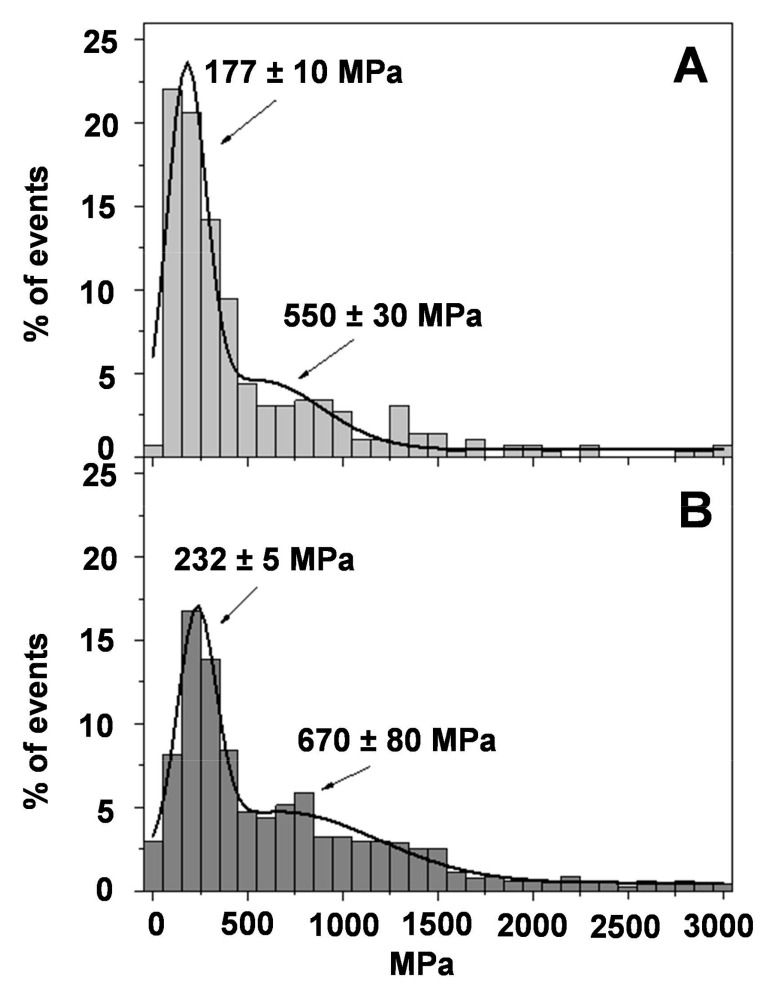
Young’s moduli distribution obtained by means of AFM Force Spectroscopy; (**A**) burned skin treated with commercial reference; (**B**) burned skin treated with PLX/CS/HA-VM. Continuous line is the fitting of the experimental data to normal distributions.

Figure 9A shows the effect of the commercial reference formulation silvederma^®^ on the skin. The recovering of the main peak of the Young′s modulus in the region 100−200 MPa can be observed, as in Figure 7A. Moreover, the second peak is also close to that observed in healthy skin at 570 MPa. The effect of PLX/CS/HA-VM, which is depicted in Figure 9B, is of great interest. The main peak observed in healthy skin is recovered, although with a lower frequency value (17%) and a little bit higher as it is centered in the region of 100–300 MPa. It is also possible to observe that the distribution of the Young′s modulus at high values is decreased and a second peak seems to appear close to the 670 MPa. It is not far away from that observed in healthy skin or the skin treated with the commercial reference silvederma^®^. It is important to remember that PLX/CS/HA-V (Figure 8B) and PLX/CS/HA-M (Figure 8C) were not capable of recovering the elasticity profile of healthy skin, but the combination of both vitamins and MLT seems to be able to reach more than a significant recovering of the surface properties of natural rat skin.

## 4. Discussion

In previous studies [17,36], we evaluated separately the effect of vitamins A, D, E and MLT formulated in hydrogel of PLX, CS and HA aimed for skin wound healing. They evidenced great healing properties on induced skin burns, while being biocompatible with healthy skin. In the present study, we go a step further and investigate the effect of the combination of both vitamins A, D, E and MLT by using the previously developed hydrogel. Hydrogels are 3-D cross-linked polymeric networks with the ability to uptake a high amount of water or biological fluid [50]. In this context, the physicochemical properties of this formulation revealed a temperature dependant behavior due to PLX. Below gelation temperature, the formulation was in sol state (Figure 1A). PLX is a non-ionic tri-block copolymer of hydrophilic polyethylene oxide and hydrophobic polypropylene oxide. At a low temperature, the polymer molecules are encircled by a hydration layer in aqueous solution. However, as the temperature rises, the rupture of hydrogen bonds between solvent and hydrophilic chains takes place, and therefore hydrophobic interaction between polyoxypropylene units increases, leading to gel formation (Figure 1B) [51]. This circumstance is particularly interesting for administration purposes because it could be applied as sol state in the affected skin area and become a gel at physiological temperature, avoiding rubbing, pain or contamination and making the adherence to the wound easier.

On the other hand, the pH of topical formulations plays an important role with significant implications [52]. The pH of PLX/CS/HA-VM was 5.0 ± 0.1. This value can be considered an optimal value considering that the pH of healthy skin is around 4.7 [53]. This acidic medium provides a defense barrier against bacterial expansion and improves fibroblast growth [54]. Finally, the internal structure of PLX/CS/HA-VM, as shown in Figure 2, provides an interconnected channel structure, which was probably caused by the cubic self-assembly of micellar PLX [38]. It can serve as a reservoir for exudates. Rough surfaces may be caused by the action of CS [55].

The use of antioxidant actives such as vitamins A, D, E and MLT was aimed to counter the consequences of oxidative stress at the wound’s surface. The combination of MLT and vitamins suggests a synergistic effect. The antioxidant activity of PLX/CS/HA-VM in terms of RSA was around 70% (Figure 3). This antioxidant activity has been demonstrated to provide a clear beneficial effect on the wound healing process by regulating the overproduction of ROS [56]. In the same context, the antibacterial activity of PLX/CS/HA-VM is also an important factor for skin wound healing, because bacterial infections can increase exudates formation and delay the wound healing process. Furthermore, the reduction in pathogens at the wound area also avoids the inflammatory response [57]. As depicted in Table 1, PLX/CS/HA-VM possessed antibacterial action similar to the commercial reference silvederma^®^ containing silver sulfadiazine, except for *E. coli*. This action might be due to ingredients such as PLX and CS, whose antimicrobial properties have been previously described [27,58], and active ingredients such as MLT, which possesses intracellular chelator action. Despite these results, this antimicrobial action cannot be considered as an antibiotic agent, but it demonstrated huge potential to efficiently prevent the wound from bacterial infection and, thus, might provide a synergistic effect, acting like a disinfectant to the wound healing action of vitamins.

The wound healing performance of PLX/CS/HA-VM was further investigated by in vivo tests (Figure 4). As the wound healing process involves extensive oxidative stress to the system, an antioxidant cocktail was applied for promoting skin wound healing. There were no statistically significant differences in wound healing efficacy in terms of percentage of wound reduction after 14 days between PLX/CS/HA-VM and silvederma^®^. The latter is a topical antibiotic used in partial thickness and full thickness burns to prevent infection. This event revealed the wound healing potential of PLX/CS/HA-VM objectively. For a deeper analysis, histological studies were accomplished. It was observed that skin treated with PLX/CS/HA-VM showed less infiltration of inflammatory cells, smaller epidermal thickness and more epidermal appendices when compared to the commercial reference treatment (Figure 5). These results were in line with other studies reporting the wound healing potential of vitamins and MLT. However, apart from their antioxidant properties, the mechanisms of actions through which assayed vitamins exert the healing action are confusing and controversial [59,60,61]. On the other hand, antiapoptotic and p53-inhibitory processes seem to be involved in the healing mechanism of MLT [62].

In the present study, the possibility of using AFM as a complementary technique to evaluate the skin properties after induced burns was also investigated. It was found that damaged skin showed higher Young’s modulus values, and this is indicative of stiffer regions. As expected, after burn heat exposition, the tissue could present an accumulation of liquids that rigidify the tissue, and although the liquid is progressively drained, it could remain in the wound for weeks. It is also possible that there was an increase in the epidermal thickness due to the wound caused by the burn, as it was observed in the histological frames. When PLX/CS/HA-V was applied on burned skin, no significant changes were observed. The distribution of Young′s modulus values was quite similar to that of untreated skin or the skin treated with PLX/CS/HA. It was surprising that in previous investigations [17] the hydrogel containing vitamins A, D and E showed good results on healing burn wounds. It is worth mentioning here that AFM is a surface microscope and it can infer information about the surface of the skin or how the region beneath this surface can modify its properties. This means that previous results are not contradictory with these found now. In fact, the tissue heals from the bottom to the surface, so the surface is the last region to be restored. The vitamins used are soluble in organic solvents as evidenced by their log *p* values, which are 5.68, 7.50 and 12.2, for vitamin A, D and E, respectively [63]. Therefore, these vitamins are quite hydrophobic, and they could easily permeate through the stratum corneum to reach the epidermis where the healing of the tissue could start. If vitamins are not retained in the skin’s surface, it is possible that the mechanical properties of burned skin would not have been restored, at least at the nanoscale.

Conversely, a different result was observed in the case of MLT. Previous studies showed the goodness of the formulation and the Young’s modulus analysis also evidenced this effect. The partial recovering of the Young′s modulus profile revealed that PLX/CS/HA-M was effective in the healing process of the wound (Figure 8C), or at least the surface of the skin and the surrounding area, as the skin treated with the PLX/CS/HA-M partially recovered the elastic properties of normal skin. MLT is a low water-soluble molecule, but its log *p* value is 1.18 [63]. Making a comparison between MLT and vitamins, it is possible that MLT molecules might remain in a higher concentration in the stratum corneum, promoting the healing of the wound. It is also presumed that MLT could permeate through the stratum corneum in a higher proportion; this can allow it to reach the epidermis layer.

To go deeply into the treatment of burned rat skin, the commercial reference formulation silvederma^®^ was compared with the hydrogel loading antioxidant cocktail PLX/CS/HA-VM. The observed effect of PLX/CS/HA-VM was of great interest. In this case, the recovering of the Young’s modulus profile indicates that the formulation is effective in healing the wound, or it at least shows comparable efficacy to skin treated with silvederma^®^. This correlates well with the histological results, where epidermal thickness was reduced, with less infiltration of inflammatory cells. The wound healing effect of PLX/CS/HA-VM could be due to the combination of the regeneration of the epidermis (vitamins and MLT) and the stratum corneum (MLT).

## 5. Conclusions

In this work, we demonstrated the significant wound healing effect of an antioxidant cocktail composed of vitamins A, D, E and MLT loaded in thermo sensitive hydrogel for the treatment of skin wounds. The formulation showed antibacterial, antioxidant action and enhanced the wound healing process in burned rat skin. In addition, in vivo and histological studies AFM Force Spectroscopy was employed for the analysis of the mechanical properties of rat skin. The combination of AFM with other common techniques such as histology or oxidative stress could improve understanding of the healing processes of the skin.

## Figures and Tables

**Table 1:** Inhibitory halos and growth reduction produced by PLX/CS/HA, PLX/CS/HA-VM and the reference formulation silvederma^®^ against different pathogen microorganisms.

Microorganisms	Inhibition Halos (mm)			Growth Reduction		
	Reference	PLX/CS/HA	PLX/CS/HA-VM	Reference	PLX/CS/HA	PLX/CS/HA-VM
*Acinetobacter baumanii* ATCC 19606	7 *	8	8	+ *	+	+
*Acinetobacter baumanii* ABAU	15	10	9	+	+	+
*Escherichia coli* ATCC 25922	7	0	0	+	−	−
*Pseudomonas aeruginosa* ATCC 27823	7	10	7	+	+	+
*Pseudomonas aeruginosa* PAO-1	7	8	7	+	+	+
*Staphylococcus aureus* ATCC 29213	7	9	9	+	+	+
*Staphylococcus aureus* MARSA ATCC 43300	7	11	11	+	+	+
*Candida albicans* ATCC 10231	7	9	9	+	+	+

(*) Observed resistant colonies; (−) no growth inhibition; (+) growth inhibition.

## Data Availability

No new data were created or analyzed in this study. Data sharing is not applicable to this article.
